# Vat Polymerization 3D Printing of Hydrogels With Tunable Porosity

**DOI:** 10.1002/smtd.70988

**Published:** 2026-08-28

**Authors:** Liwei Liu, Liwen Zhang, Xumin Huang, Yuan Xu, Jason R. Stokes, Xinyan Gu, Naufal Kabir Ahamed Nasar, Xiaoming Sun, Jiali Zhai, Thomas P. Davis, Ruirui Qiao

**Affiliations:** ^1^ Australian Institute for Bioengineering and Nanotechnology The University of Queensland Brisbane Queensland Australia; ^2^ School of Chemical Engineering The University of Queensland Brisbane Queensland Australia; ^3^ Australian National Fabrication Facility Queensland Node (ANFF‐Q) The University of Queensland Brisbane Queensland Australia; ^4^ School of Science, STEM College RMIT University Melbourne Victoria Australia

**Keywords:** 3D printing, cell proliferation, macroporous hydrogel, soft materials, tissue engineering

## Abstract

Three‐dimensional, interconnected hydrogel networks are central to tissue engineering and disease modeling, where tailored pore architecture and mechanical robustness are essential for supporting cellular functions. However, the limited ability to engineer microstructural features in vat polymerization 3D‐printed natural hydrogels often compromises scaffold performance, as oversized pores reduce cell attachment and cell–cell interactions while smooth pore walls lack essential topographical cues. Here, we report an emulsion‐based ink for vat polymerization 3D printing that enables the fabrication of hydrogels with finely tunable and highly interconnected porous architectures. An oil‐in‐water resin formulated using gelatin methacrylate (GelMA) contains stable solvent nanodroplets that act as sacrificial templates during photopolymerization. Removal of the dispersed phase yields additive‐free porous hydrogels with pore sizes ranging from 0.66 to 46.15 µm and a 2.5‐fold enhancement in compressive toughness. This strategy is compatible with digital light processing (DLP) and broadly applicable to multiple photocurable biopolymers, including alginate methacrylate (ALMA) and hyaluronic acid methacrylate (HAMA). The resulting porous scaffolds promote enhanced cell attachment, proliferation, and cell–cell interactions, highlighting the potential of this vat polymerization‐compatible platform for advanced biofabrication.

## Graphical abstract

Nanodroplet emulsion templating enables DLP printing of porous hydrogels with pore sizes tunable across two orders of magnitude, from 46.15 to 0.66 µm. The printed interconnected networks retain high fidelity while improving mechanical robustness and cellular responses, providing a versatile platform for porous hydrogel biofabrication.

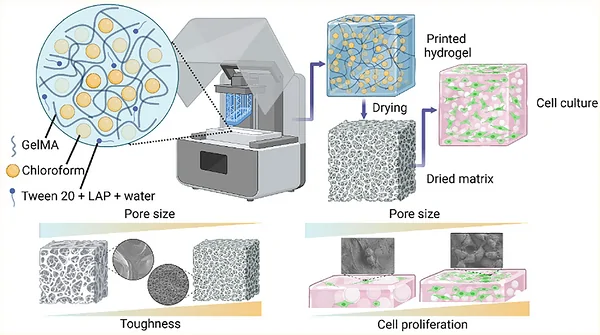

## 1 Introduction

Three‐dimensional (3D) printing has emerged as a revolutionary strategy for engineering hydrogel scaffolds, offering a precise spatial control over the architecture and thus the ability to fabricate structures customized for emulating the extracellular matrix (ECM) [1, 2]. Central to this capability is the development of photopolymerizable biopolymers such as gelatin methacrylate (GelMA) and alginate methacrylate (ALMA). Upon photoinitiation, the methacrylate groups undergo radical polymerization, resulting in the irreversible formation of a spatially defined hydrogel network during printing [2, 3].

This light‐triggered polymerization is intrinsically compatible with vat polymerization‐based 3D printing technologies, including stereolithography (SLA) and digital light processing (DLP), distinguishing methacrylated biopolymers from conventional hydrogel systems that rely on slower or condition‐limited crosslinking mechanisms. Moreover, unlike purely synthetic photo‐crosslinkable polymers (e.g., polyacrylamide derivatives), GelMA and ALMA retain intrinsic bioactivity and enzymatic degradability, thereby integrating high‐resolution printability with cell‐instructive functionality [4]. This unique combination of photopolymer chemistry and additive manufacturing has positioned methacrylated biopolymers as the key promising candidate for the next‐generation hydrogel scaffolds in tissue engineering.

Despite these advantages, a persistent challenge in the biomedical application of methacrylated biopolymers is the limited ability to tailor hydrogel microarchitecture in a systematic manner [5]. In 3D‐printed hydrogels, pore size and pore size distribution critically regulate nutrient transport, biomolecule diffusion, and cell–matrix interactions. Excessively large pores (>150 µm) reduce surface‐to‐volume ratio, weaken integrin‐mediated adhesion, impair early cell anchorage, and disrupt long‐range cell–cell interactions required for coordinated tissue organization [6, 7]. Furthermore, the smooth pore walls generated under such conditions lack microscale roughness, depriving cells of the topographical cues required for focal adhesion formation and cytoskeletal alignment, thereby constraining proliferation and migration [8, 9, 10]. These findings underscore that the performance of hydrogels in tissue engineering is not solely governed by the stiffness or chemistry in bulk scale, but also by a delicate interplay between pore‐scale microarchitecture and cell‐instructive signaling [11, 12].

Numerous strategies have been explored to regulate hydrogel pore size, including modulation of polymer concentration [13], or crosslinking density [14], freeze‐drying [15], gas foaming [16], and phase separation [17]. However, these approaches often couple pore formation with changes in bulk mechanical properties, limiting their applicability in scenarios where specific microstructural features are desired without altering macroscopic functionality. Porogen leaching [18] and sacrificial templating techniques [19, 20] have also been explored, yet they generally introduce structural heterogeneity, require complex processing steps, or compromise resolution when integrated with 3D printing.

More recently, two‐phase emulsion templating has emerged as a versatile strategy for introducing porosity into both natural and synthetic hydrogels. By exploiting the immiscibility between aqueous and organic solvent phases, emulsions can comprise stable droplets that function as a dynamic template during polymerization [12]. In GelMA systems, this method typically yields pores ranging from ca. 100 µm to 1 mm in diameter, with some formulations achieving smaller pore size of 24.8–58.7 µm [21, 22, 23]. However, because the immiscible phase is generally of more viscous than water and has a higher molecular weight, the emulsification leads to a size distribution of droplet biased toward dimensions in microns or several tens of microns, yielding a comparable pore size distribution in the network of resultant hydrogels [24, 25]. While this strategy provides an important step forward, it remains limited in its ability to generate pores consistently at the submicron to low‐micron scale that are increasingly recognized as essential for recapitulating ECM‐like microenvironments. Achieving this level of structural control is further complicated by the need to maintain mechanical integrity and the fine feature resolution in 3D‐printed scaffolds, which are requirements that existing pore‐forming methods struggle to meet.

Here, we report a vat polymerization 3D printing strategy that overcomes these limitations, enabling the fabrication of porous hydrogels with pore sizes finely tunable from 46.15 down to 0.66 µm without compromising structural integrity. This is achieved through a two‐phase emulsification approach that disperses an organic solvent into the GelMA solution, yielding stable oil‐in‐water emulsions with nanodroplets lasting for up to 24 h at room temperature. Remarkably, these nanodroplets are preserved during DLP photopolymerization and become uniformly embedded in the hydrogel matrix in a layer‐by‐layer manner. The process enables the tuning of pore size while maintaining print resolution and mechanical robustness, without the need for pore‐forming post‐processing. The resulting scaffolds exhibit highly interconnected networks that facilitate the diffusion of nutrients and provide topographical cues essential for cell anchorage and organization, leading to 116% and 71.04% increase in proliferation of endothelial hCMEC/D3 and cancer MDA‐MB‐231 cells, respectively, by day 5 relative to the controls. Importantly, this emulsification‐based strategy is universal across a broad range of hydrogels made from photo‐crosslinking polymers, including ALMA, HAMA, and GelMA–alginate hybrids. By enabling the tailoring of pore architecture irrespective to the polymer chemistry and concentration, it establishes a generalized and up‐scalable route for engineering porous scaffolds that retain both high print fidelity and tunable mechanical properties. The wide applicability of this technique underscores its potential as a platform for 3D printing functional hydrogel‐based materials in tissue engineering, which requires various materials with diverse properties to replicate the biochemical and mechanical functionality of different tissue microenvironments.

## 2 Results and Discussion

### 2.1 Preparation and Characterization of 3D Printing GelMA Emulsion Resin

GelMA, one of the most extensively studied and commercialized naturally derived polymers for cell culture models, was selected as the model system to validate our novel two‐phase emulsification method [26, 27]. The emulsion resin was prepared by sonication of an aqueous GelMA‐based resin with chloroform, dispersing the organic solvent into uniformly sized nanodroplets [28]. The emulsion resin was subsequently applied in a DLP 3D printer, followed by post‐processing to fabricate macroporous hydrogels (Figure 1a). GelMA‐based resin was prepared by 7.5% (w/v) synthesized GelMA as monomer (Figure S1), 2% (w/v) polyethylene glycol (PEG), 0.5% (w/v) lithium phenyl‐2,4,6‐trimethylbenzoylphosphinate (LAP) as photoinitiator. Additional 2% (w/v) PEG was added to GelMA resin to modestly enhance mechanical robustness and improve handling of the GelMA constructs. Surfactants and chloroform were subsequently introduced, followed by sonication to generate the emulsions. Because surfactants play an essential role in stabilizing nanodroplets during light‐assisted vat polymerization, two common agents—cetrimonium bromide (CTAB) and Tween‐20—were evaluated. The 2% (v/v) Tween‐20 (relative to water) proved most effective, preventing the destabilization and maintaining a consistent nanodroplet size, irrespective to the sonication time (5 and 10 min, see Figure S2 and Table S1). Moreover, the physical properties of organic phase also exert a critical influence on droplet formation and stability [29]. Chloroform was selected as the dispersed phase due to its low miscibility with water, which enhances droplet stability, and high volatility, which enables efficient removal after polymerization [30]. Different chloroform‐to‐aqueous phase ratios, ranging from 1:10 to 1:1, were investigated to optimize the formulation for 3D printing. As shown in Figure S3, increasing the solvent ratios to 4:10, 5:10, and 1:1 causes the droplets to progressively approach the packing limit, resulting in increased coalescence and pronounced phase separation. By contrast, lower solvent ratios (1:10 to 3:10) yielded stable emulsions. Accordingly, emulsion resins with lower solvent ratios of 1:10, 2:10, and 3:10 were used for subsequent experiments and are hereafter referred to as Groups 1, 2 and 3, respectively, while the resin without solvent was designated as the control (Table S2).

**FIGURE 1:**
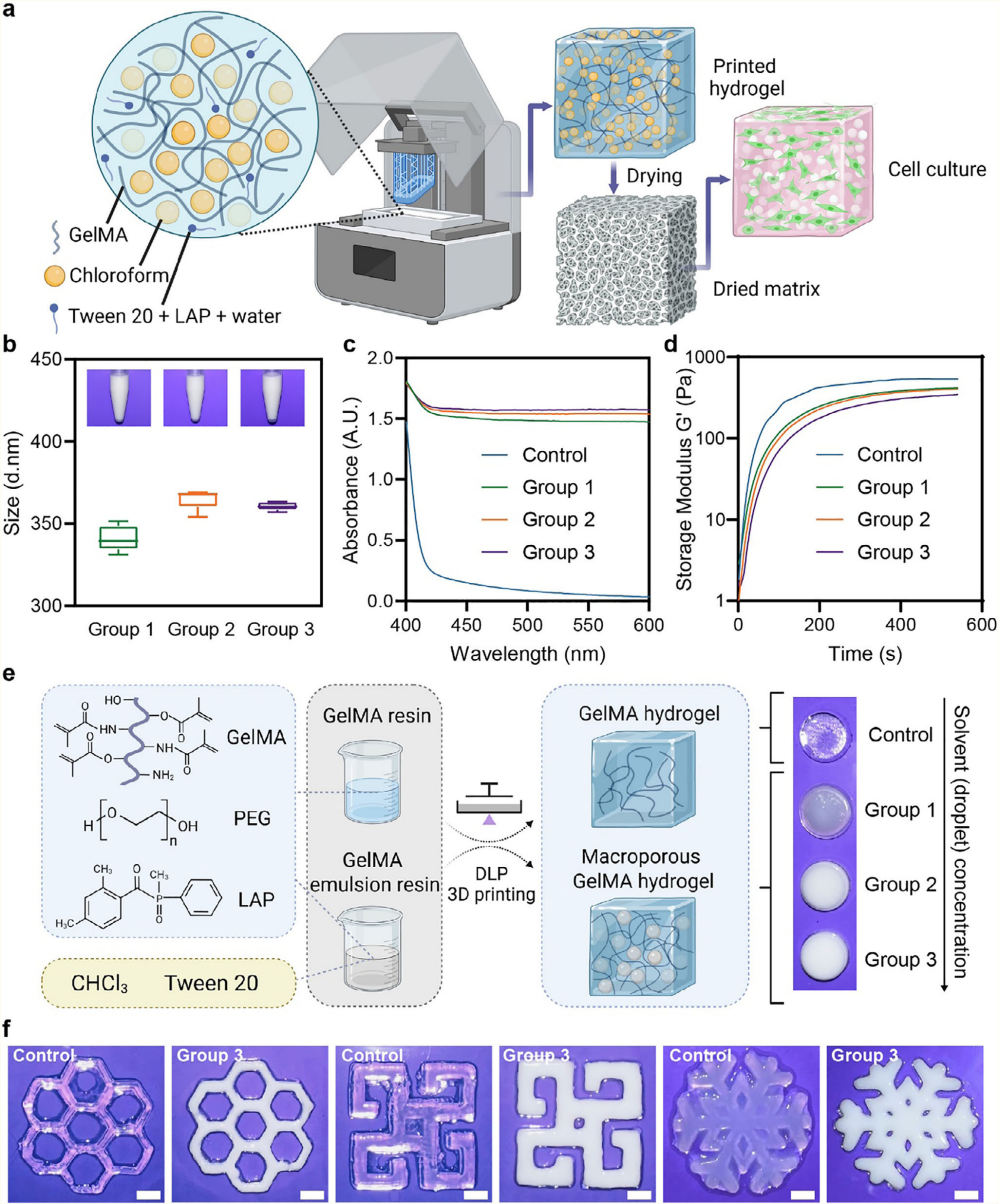
(a) Schematic illustration of the preparation of 3D‐printed macroporous hydrogel scaffold with GelMA emulsion resin. (b) Nanodroplet size distribution of resins with varying volume ratios of organic solvent (chloroform:GelMA solution = 1:10, 2:10, and 3:10, v/v), referred to as Groups 1, 2 and 3, respectively; inlet shows their corresponding photographs taken 20 min after sonication. Data are presented as mean ± standard deviation (SD) (*n* = 3 independent samples). Error bars represent SD. (c) UV‐vis spectrum of GelMA resins with different volume ratios of organic solvent, conventional GelMA resin is denoted as control. (d) In situ photopolymerization kinetics of emulsion resins with varying volume ratios of organic solvent toward hydrogel formation after gelation point. (e) Schematic illustration of the formulation and the DLP 3D printing process with conventional GelMA and novel GelMA emulsion resin, with photographs of 3D‐printed objects showcasing printability (right). (f) Complex structures printed using conventional GelMA resin (Control) and GelMA emulsion resin (Group 3). Scale bar: 5 mm.

Dynamic light scattering (DLS) revealed a relatively monodisperse droplet population across Groups 1–3, with sizes averaging between 341.2 ± 7.75 and 365.3 ± 6.31 nm, as shown in Figure 1b. These nanodroplets are stable with the size distribution remains consistent over 24 h, as shown in Figure S4. Notably, the dispersed nanodroplets impart optical turbidity to the resin, giving it an opaque appearance (Figure S3). The UV‐vis spectra (Figure 1c) reveal higher absorbance with increasing solvent ratio, reflecting increased turbidity and reduced light penetration due to higher droplet content in the system. To further investigate the effect of this increased absorbance on curing, the gelation under light irradiation (λ = 405 nm, 1.68 mW cm^−^^2^) was monitored through rheological measurement. Although nanodroplets attenuated UV transmission, they did not significantly alter gelation kinetics (as seen from the slope of change in storage modulus vs. time, in Figure 1d). Collectively, these results demonstrate that incorporating nanodroplets yields resins with excellent stability without compromising 3D printing performance.

To investigate and optimize the formation of macropores in hydrogels, these three emulsion resins with different volume ratios of solvent phase were subsequently applied to DLP 3D printer to obtain three groups of 3D‐printed hydrogels (Group 1, Group 2, and Group 3), as illustrated in Figure 1e. The printing parameters were set with a layer thickness of 0.05 mm and an exposure time of 10–30 s per layer (Table 1). Importantly, the incorporation of nanodroplets into the resins did not compromise the resolution and accuracy of the 3D‐printed hydrogels. Even at the highest solvent content, cylinders printed from the emulsion resin exhibited diameters of 10.37 ± 0.10 mm and thicknesses of 3.06 ± 0.01 mm, comparable to those obtained using the conventional resin (10.27 ± 0.11 mm and 3.06 ± 0.04 mm, respectively; Table 1). Although the color shifted from transparent to white, the 3D‐printed macroporous hydrogel maintained excellent shape fidelity, characterized by sharply defined edges and smooth, defect‐free surfaces (Figure 1e). Additionally, three architecturally complex structures were fabricated using the Group 3 emulsion resin, which our prior results identified as exhibiting the strongest attenuation of photopolymerization kinetics among all samples. The resulting constructs maintained excellent structural precision and smooth surface finish (Figure 1f and Figure S5), underscoring its remarkable printability and its capacity to reliably produce geometrically intricate architectures. Overall, these findings establish a facile and robust strategy for formulating nanodroplet‐containing resins that exhibit high emulsion stability and photopolymerization efficiency while maintaining geometric accuracy.

**TABLE 1:** Printing parameters and geometry of 3D‐printed hydrogels using GelMA emulsion resin.

Group	Solvent: aqueous solution (v/v)	3D‐printing parameter		3D‐printed object	
		Curing time/layer (s)	Thickness/layer (mm)	Diameter (mm)	Thickness (mm)
Control	—	10	0.05	10.27 ± 0.11	3.06 ± 0.04
1	1:10	15	0.05	10.23 ± 0.08	3.01 ± 0.02
2	2:10	20	0.05	10.32 ± 0.13	3.03 ± 0.04
3	3:10	30	0.05	10.37 ± 0.10	3.06 ± 0.01

### 2.2 Fabrication and Characterization of 3D‐Printed Macroporous Hydrogel

During the 3D printing process, the GelMA continuous phase was photopolymerized into a crosslinked network, while the dispersed chloroform nanodroplets were entrapped within the matrix. Subsequent removal of volatile solvent droplets generated well‐defined voids, yielding a macroporous architecture through a droplet‐templating mechanism that provided a highly porous structure and interconnected channels. To validate, scanning electron microscopy (SEM) was employed to examine the macropore size and distribution within the 3D‐printed hydrogels. As the solvent content of the emulsion increased, the resulting hydrogels exhibited progressively greater interconnectivity, accompanied by smaller and more densely packed pores (Figure 2a). Notably, the pores exhibited increasing homogeneity as the solvent content increased, adopting a uniform and rounded shape in Group 3. Simultaneously, the structural framework became progressively finer, as the expanding interfacial area caused the continuous phase to thin and envelop the dispersed solvent phase [31]. Analysis of pore size from SEM images [32] demonstrated a reduction in pore diameter from 46.15 ± 16.66 µm in control to as small as 0.66 ± 0.18 µm in Group 3 (Figure 2b).

**FIGURE 2:**
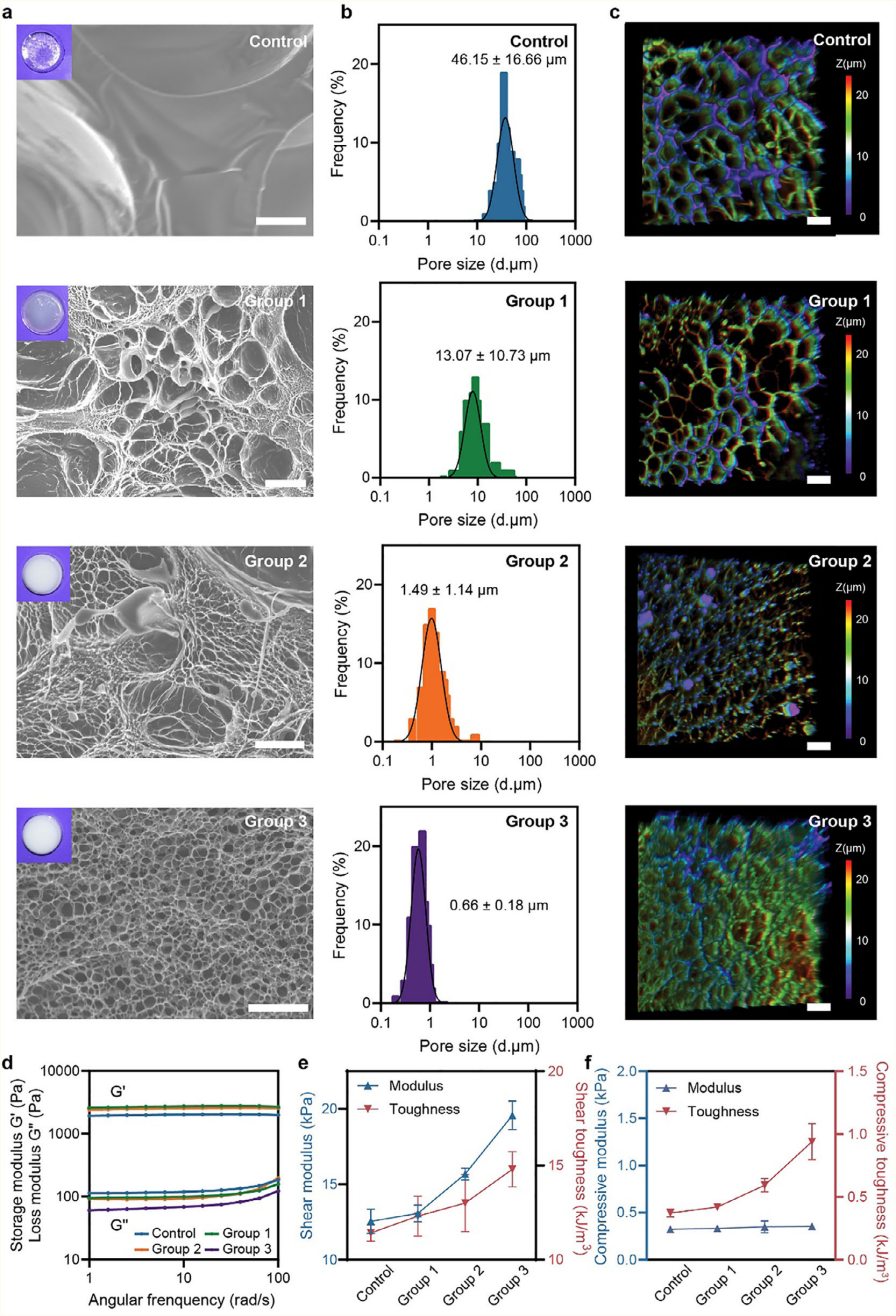
Morphological and mechanical characterization of 3D‐printed macroporous GelMA hydrogel. (a) SEM images of lyophilized 3D‐printed macroporous hydrogel with different volume ratios of solvent, which control denotes the samples printed using conventional GelMA resin. Scale bar: 10 µm. (b) Pore size distribution determined from SEM image analysis (*n* = 100 pores measured). The annotated value represents the mean pore diameter ± SD. (c) 3D reconstruction confocal images of the lyophilized macroporous hydrogel with color‐coded depth. Scale bar: 10 µm. (d) Dependence of storage modulus (G′) and loss modulus (G″) on the frequency of oscillation. (e) Shear modulus and toughness, and (f) compressive modulus and toughness of 3D‐printed macroporous hydrogel with different volume ratios of solvent. Data represents the mean ± SD (*n*  = 3 independently printed hydrogel), error bars indicate SD.

To verify the morphological changes, 3D‐printed macroporous hydrogels were stained using rhodamine B and visualized using confocal laser scanning microscope (CLSM), followed by 3D reconstruction of the acquired z‐stack images. The reconstructed data was consistent with the SEM observations, showing that hydrogels exhibited progressively denser interconnections, smaller pore sizes, and more uniform pore sizes with increasing solvent content (Figure 2c). This trend can be ascribed to the suppression of coalescence at elevated dispersed phase volume, which effectively limits droplet growth and leads to the formation of smaller, more monodisperse pores upon photopolymerization [33]. Conversely, a low droplet density compromises stability by facilitating Ostwald ripening, which accelerates mass transfer from small to large droplets, and produces a broad, polydisperse pore‐size distribution with inhomogeneous morphology [33]. Under DLP processing, the emulsion further undergoes continuous spatiotemporal evolution driven by layer‐by‐layer resin redistribution and concurrent network formation, [34] promoting nanodroplet deformation, close packing, and dynamic rearrangement into an interconnected architecture. Consequently, while increasing chloroform content consistently resulted in smaller pore sizes, the final pore architecture is influenced by multiple factors beyond the initial droplet dimensions, including emulsion stability, photopolymerization, and post‐processing [35, 36].

Importantly, unlike established fabrication strategies that generally yield hydrogels with pores in a relatively narrow size distribution (Figure S6 and Table S3), our method offers superior flexibility, enabling systematic engineering of pore sizes spanning 46.15 to 0.66 µm. Moreover, the increased porosity was accompanied by enhanced swelling, with Group 3 exhibited the highest swelling ratios after 72 h in both deionized water (2524%) and PBS buffer (2261%) (Figure S7 and S8). Compared with the control GelMA hydrogels, these values corresponded to increases of 182% and 145%, respectively, which can be attributed to the higher pore density and greater capacity for fluid uptake. In addition, 3D micro‐CT reconstruction of the swollen Group 3 hydrogel revealed an interconnected pore network throughout the matrix, as confirmed by segmentation of the pore phase (Figure S9).

### 2.3 Tunable Mechanical Properties of Macroporous Hydrogel

Following the successful fabrication of macroporous hydrogels, the mechanical properties were evaluated using shear and compressive deformation tests. The oscillatory stress sweep analysis demonstrated a predominance of elastic (gel‐like) behavior, with the storage modulus (G′) exceeding the loss modulus (G″) within the linear viscoelastic region (LVR; Figure S10). The LVR (where G′ and G″ are independent on amplitude) for macroporous hydrogels (Group 1–3) in general extends to larger strains relative to the control sample, indicating a higher resistance to the nonlinear deformation. Within the LVR, all gels exhibited comparable values of G′ and G″, at least in logarithm scale irrespective of their composition. The G′ and G″ are largely independent of frequency, suggesting a classical behavior seen in most gels consisting of a stable network upon the imposed oscillatory deformation (Figure 2d). Note that the control hydrogel exhibited a lower G' compared to macroporous gels. This observation indicates that the incorporation of nanodroplets has a strengthening effect on the storage modulus (elasticity) of the hydrogel, which we attribute to the restricted relaxation of the gelling network caused by the voids/macropores.

To further investigate the response of these hydrogels upon a nonlinear deformation, macroporous hydrogels were subjected to shear (shear rate of 0.01/s) and uniaxial compression (crosshead speed 1.1 mm/min) tests to failure. The shear modulus (determined from the initial linear part of the stress–strain curve) of hydrogels increased from 12.55 ± 0.65 to 19.56 ± 0.77 kPa, as the solvent content increased (Figure 2e and Figure S11). Shear toughness (defined as the area under the stress–strain curve before the fracture) also rose from 11.42 ± 0.35 to 14.79 ± 0.76 kJ/m^3^ with increasing solvent content, for the testing specimens. Upon a compressive deformation, the compressive modulus, defined as the initial slope between 1 and 4% strain, for all tested hydrogels are comparable at 0.34 ± 0.03 kPa (Figure 2f). This invariance reflects that small strain stiffness is governed primarily by the base polymer concentration and the crosslink density, both of which were held constant across formulations and were independent of pore formation [37]. However, hydrogels prepared from emulsions with higher solvent contents yielded and fractured at larger compressive strains, resulting in a 2.5‐fold increase in compressive toughness, from 0.37 ± 0.02 kJ/m^3^ for Group 1 to 0.94 ± 0.11 kJ/m^3^ for Group 3 (Figure 2f and Figure S12). In general, smaller pores and a more uniform microstructure provide continuous load paths that redistribute stress and dissipate energy, thereby supporting greater load‐bearing capacity [38, 39, 40]. Conversely, increasing pore size can reduce the effective load‐bearing area and amplify the stress concentration (which ultimately leads to the fracture) [41]. We thus consider that the same principle should apply here that macroporosity created from nanodroplets substantially enhances the toughness and strength of 3D‐printed macroporous hydrogels without notably increasing the stiffness [42].

Integrating these findings, the Group 3 hydrogel exhibited the most promising mechanical properties, i.e. the greatest mechanical strength due to its highly interconnected network and uniformly distributed pores. Consequently, it was selected for subsequent studies and designated as the macroporous GelMA hydrogel (m‐GelMA), while the control hydrogel was denoted as GelMA.

### 2.4 Extension of Nanodroplet‐Templated Printing in General Hydrogel Systems

In addition to gelatin, natural polymers such as alginate and hyaluronic acid are widely used in bioengineering applications owing to their superior biocompatibility [43]. To demonstrate the versatility of our approach, alginate‐ and hyaluronic acid‐based, as well as GelMA‐alginate hybrid macroporous hydrogels were assessed for nanodroplet formation, printability, and mechanical properties. Following the workflow for GelMA fabrication, alginate and hyaluronic acid were functionalized with methacrylate groups, yielding ALMA and HAMA (Figure S13 and S14). A GelMA–alginate hybrid formulation (GelMA–Alg) was also prepared. Proposed method was then applied (solvent: aqueous solution 3:10, v/v) to generate nanodroplet‐based resins under the probe sonication, which were then used to fabricate macroporous hydrogels via photopolymerization (Figure 3a and Table S2).

**FIGURE 3:**
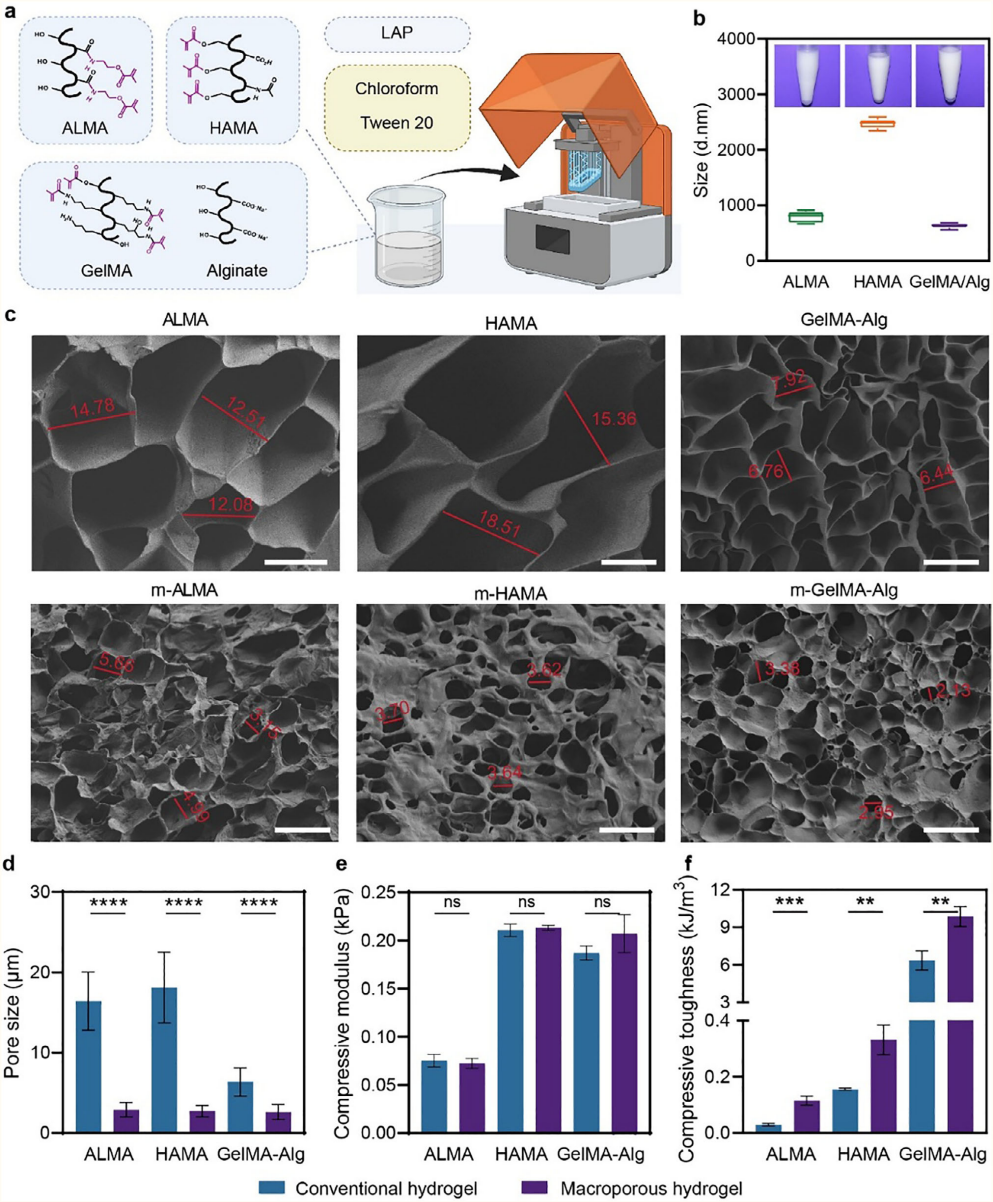
Characterization of 3D‐printed macroporous hydrogels. (a) Schematic illustration of the formulation with different resins (ALMA, HAMA and GelMA‐Alg). (b) Nanodroplet size distribution of different resins with 3:10 solvent: resin ratio; insets show their corresponding photographs taken 20 min after sonication. Data represents the mean ± SD (*n*  = 3 independent samples), error bars represent SD. (c) SEM images of lyophilized 3D‐printed ALMA, HAMA and GelMA‐Alg hydrogel without solvent (top) and with 3:10 solvent: resin ratio (bottom, denoted as m‐ALMA, m‐HAMA and m‐GelMA‐Alg). On‐plot labels report pore diameter (µm). Scale bar: 10 µm.(d) Pore size distribution analyzed from SEM images (pores measured *n*  = 100). Error bars indicate SD. The statistical significances were assessed with unpaired student's *t*‐test. (e) Compressive modulus and, (f) toughness of the conventional hydrogel and their macroporous counterparts. Data represents the mean ± SD (*n*  = 3 independent samples), error bars indicate SD. The statistical significance was assessed with unpaired student's *t*‐test.

DLS results (Figure 3b) demonstrated that the average nanodroplet sizes in ALMA and HAMA resins were 724.06 ± 64.72 nm and 2425 ± 63.64 nm, respectively. The average nanodroplet size in the HAMA resin was significantly larger than those in ALMA and GelMA resins, which can be attributed to its higher viscosity and consequent influence on droplet formation during the emulsification process (Figure S15). In general, the pronounced viscosity attenuates the shockwave from the ultrasound, thereby reducing shear and mixing that drive emulsification [28]. As a result, droplet break‐up is less efficient and the final droplet size increases in more viscous medium. Indeed, GelMA and alginate mixture (with the viscosity greater than GelMA while lower than ALMA), yields an average droplet size of 647.13 ± 16.83 nm in the emulsion prepared from the same method, sitting between the behaviors observed from using just GelMA and ALMA respectively. After 24 h, minimal coalescence was observed in any formulation (Figure S16). Overall, nanodroplets were successfully fabricated using various hydrogel resins; however, their size was dependent on the viscosity and interfacial tension of the polymer solutions [44].

Following this, these resins were employed to fabricate cylindrical macroporous hydrogels through DLP 3D printing (m‐ALMA, m‐HAMA, and m‐GelMA‐Alg) that resulted in smooth surfaces and high geometric fidelity to the design (Figure S17, Table S4). In contrast with conventional ALMA and HAMA, the use of nanodroplet‐containing resins significantly reduced pore size, revealed from SEM (Figure 3c). Quantitative analysis of these SEM images (Figure 3d) showed that, m‐ALMA hydrogels exhibited an average pore size of 2.90 ± 0.89 µm, which was substantially smaller compared to that of the reference hydrogel at 16.43 ± 3.57 µm. A similar trend was observed in m‐HAMA and m‐GelMA‐Alg hybrid hydrogels, where the pore size reduced from 18.11 ± 4.36 to 2.70 ± 0.72 µm, and from 6.34 ± 1.76 to 2.62 ± 0.94 µm, respectively. All results indicated that nanodroplet templating consistently refines the microstructure and generate macroporous structures across different hydrogel systems.

Additionally, compression tests were conducted to assess the mechanical properties of the macroporous hydrogels. In line with the observation from m‐GelMA, all m‐ALMA, m‐HAMA, and m‐GelMA–Alg hydrogels exhibited enhanced toughness while maintaining a compressive modulus comparable to their counterparts (Figures 3e,f and Figure S18). We attribute the toughness gain to a more highly interconnected load‐bearing architecture introduced by nanodroplet templating, which promotes stress redistribution and energy dissipation [39]. Since the intrinsic degree of crosslinking did not change, the compressive modulus remained similar across the different formulations [37]. These results underscore the high adaptability of the proposed technique for generating macropores in naturally derived hydrogels and confirm the substantial impact of structural modulation on mechanical enhancement, demonstrating its potential for reproducible tuning of pore architecture in application‐specific contexts.

### 2.5 Macroporous Architecture Promotes Cell Adhesion and Growth

Porous hydrogel has garnered attention in tissue engineering benefiting from its ability to mimic ECM and resemble biological scaffold [41]. However, the inherently large pore and the smooth matrix wall the GelMA hydrogel or other naturally derived hydrogel possess would often result in suboptimal cell attachment and stem cell differentiation [45]. It has been demonstrated that the mesh size and interconnectivity are strongly associated with enhanced cell proliferation across different hydrogel formulations, such as polyacrylamide and hydroxyethyl methacrylate [41, 46, 47, 48, 49]. Further, gelatin has been associated with low cell adhesion comparing to other hydrogel composition like fibrin and collagen [50].

By leveraging the scaffold's porous architecture, here we demonstrated the effect of our macroporous GelMA hydrogel with enhanced interconnectivity on the surface cell attachment and proliferation. To address potential biosafety concerns associated with residual chloroform, ^1^H NMR analysis was performed on m‐GelMA following post‐processing (Figure S19). No characteristic chloroform resonance was detected at approximately 7.26 ppm, confirming its effective removal from the hydrogel. The biocompatibility of the m‐GelMA was then examined using a CCK‐8 assay, indicating no significant toxicity to the human embryonic kidney cells HEK‐293 over a five‐day co‐incubation with the m‐GelMA (Figure S20 and S21). To assess the porous hydrogels’ ability to support cell attachment and proliferation, green fluorescent protein (GFP)‐positive human cerebral microvascular endothelial cells (hCMEC/D3) were seeded on the 3D‐printed scaffolds GelMA and m‐GelMA (Figure 4a). Cell attachment and proliferation were monitored by confocal fluorescence imaging on days 1, 3, and 5 post‐seeding, with fluorescence intensity quantified at each time point (Figure 4b,c). From day 3 to day 5, the m‐GelMA surface exhibited significantly higher fluorescence intensity than native GelMA, with increases of 57.59% and 116%, respectively. This enhanced fluorescence indicates that the macroporous architecture of the hydrogel markedly promotes endothelial cell proliferation. Live/Dead staining with quantitative analysis at day 5 further confirmed the excellent biocompatibility of both GelMA and m‐GelMA (Figure 4d,e). In line with the characteristic morphology previously described for hCMEC/D3 cells [51], high‐magnification imaging revealed that cells on the m‐GelMA surface adopted a more tightly packed, spindle‐shaped appearance than those on GelMA (Figure 4f,g), suggesting that the porous framework offers an expanded surface area that supports enhanced proliferative activity.

**FIGURE 4:**
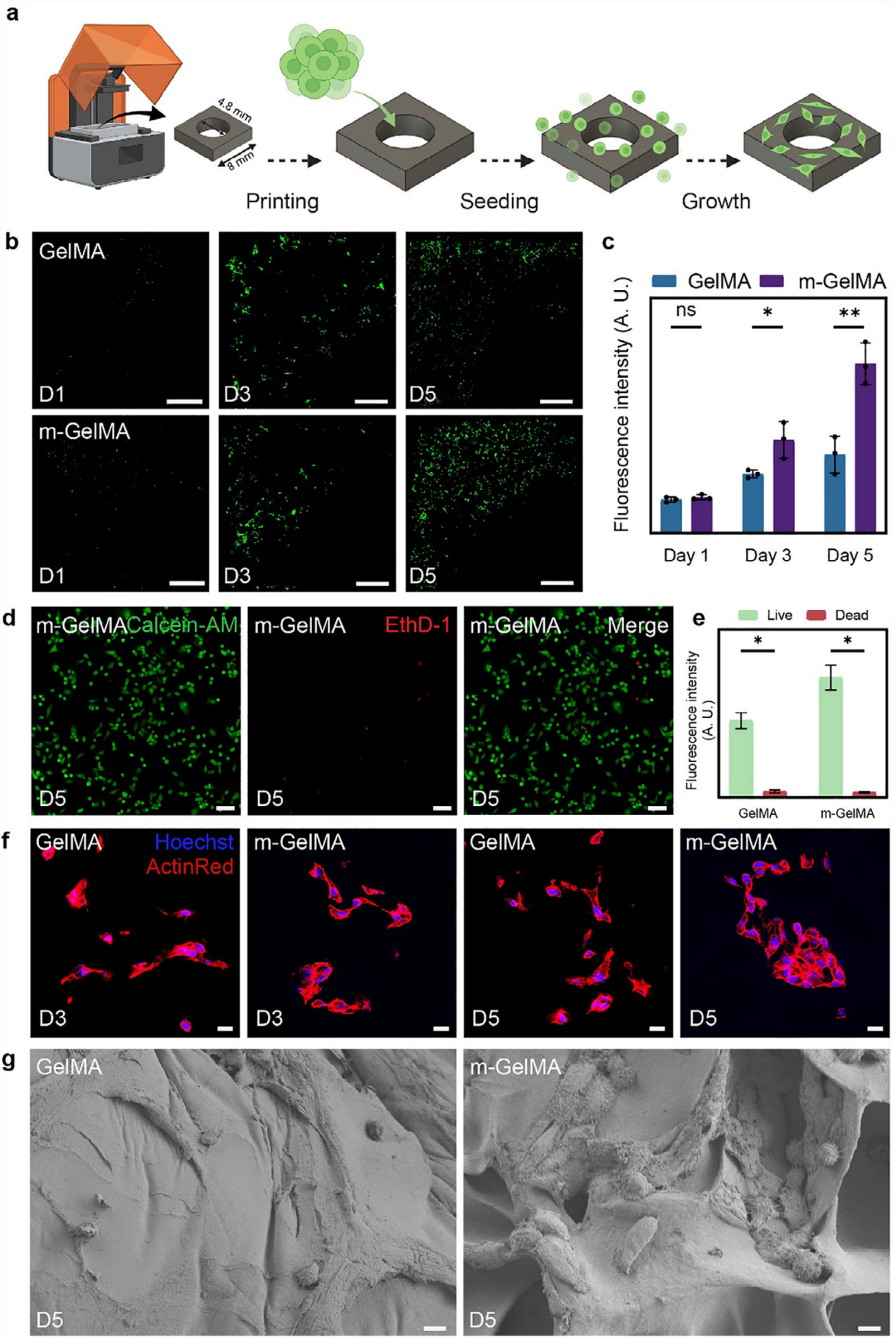
Macroporous hydrogel scaffold enhanced microvascular endothelial cell (hCMEC/D3) attachment and proliferation. (a) Schematic representation of macroporous hydrogel acts as culturing scaffold. (b) CLSM images of GFP‐positive hCMEC/D3 cells cultured on the GelMA and m‐GelMA scaffolds for 1, 3 and 5 days. Scale bar: 1 mm. (c) Fluorescence intensity of cell coverage calculated from CLSM images at different culture periods. Data represents the mean ± SD (*n*  = 3 biological replicates), error bars indicate SD. The statistical significance was examined using two‐way ANOVA with Šídák's multiple‐comparisons test. (d) Representative CLSM images of live/dead staining of hCMEC/D3 cells cultured on the GelMA and m‐GelMA scaffolds on day 5. Live cells (green) were stained with calcein‐AM, and dead cells (red) were stained with ethidium homodimer‐1. Scale bar: 100 µm. (e) Fluorescence intensity of cell coverage calculated from live/dead CLSM images on day 5. Data represents the mean ± SD (*n*  = 3 biological replicates), error bars indicate SD. The statistical significance was examined using two‐way ANOVA with Šídák's multiple‐comparisons test. (f) Representative zoom‐in CLSM images of cell morphology cultured on day 3 and 5, cells were stained with Hoechst 33342 for nucleus (blue) and counterstained with ActinRed for F‐actin (red). Scale bar: 20 µm. (g) SEM images of the cells cultured on the surface of GelMA and m‐GelMA on day 5. Scale bar: 10 µm.

To further substantiate the beneficial role of macropores in supporting cell migration and growth, we examined the attachment and proliferation of a highly motile and invasive model cell line, the triple‐negative breast cancer cells MDA‐MB‐231. Consistent with our earlier findings with the endothelial cell, the macroporous hydrogel improved initial cell adhesion by 39.40% on day 1 and subsequently promoted robust proliferation, with increases of 70.35% and 71.04% observed after 3 and 5 days of culture, respectively (Figures 5a,b). It has been reported that the MDA‐MB‐231 cells with more spread‐out and elongated features have strong adhesion and greater mobility [52]. As evidenced by CLSM close‐up images (Figure S22), the cells cultured on the m‐GelMA demonstrated abundant morphologies, characterized by large, elongated, stellate projections and prominent actin‐rich lamellipodium. In contrast, cells on GelMA displayed smaller, grape‐like morphologies, indicative of reduced cell–cell adhesion [53].

**FIGURE 5:**
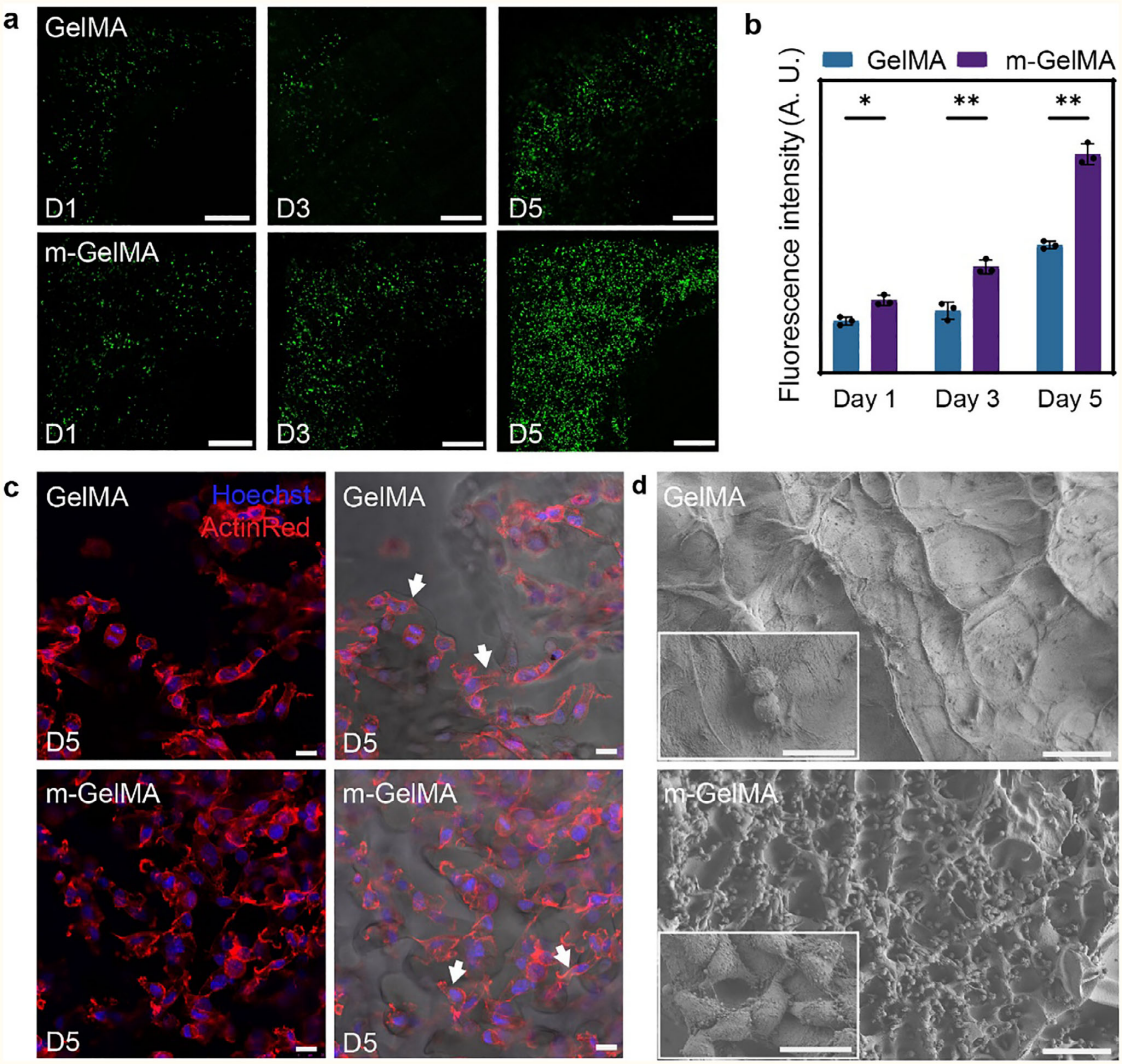
Macroporous hydrogel scaffold enhanced breast cancer cell (MDA‐MB‐231) attachment and proliferation. (a) CLSM images of GFP‐positive MDA‐MB‐231 cells cultured on the GelMA and m‐GelMA scaffolds for 1, 3 and 5 days. Scale bar: 1 mm. (b) Fluorescence intensity of cell coverage calculated from CLSM images at different culture periods. Data are presented as mean ± SD (*n* = 3 independent samples). Error bars represent SD. The statistical significance was examined using two‐way ANOVA with Šídák's multiple‐comparisons test. (c) Representative CLSM overlaid fluorescent (left) and bright field (right) images of cell morphology cultured on day 5, cells were stained with Hoechst 33342 for nucleus (blue) and counterstained with ActinRed for F‐actin (red). Arrows highlight the interaction between the neighboring cells and the matrix wall. Scale bar: 20 µm. (d) SEM images of the GelMA and m‐GelMA, and the cell cultured on their surface at day 5. Scale bar: 100 µm, inset scale bar: 20 µm.

In GelMA hydrogel, the relatively thick walls impede intercellular connectivity, whereas the reduced wall thickness and highly interconnected network of m‐GelMA generate an abundance of accessible structural binding sites, thereby enhancing cell–cell communication and facilitating cellular adhesion and migration. Superimposed fluorescence and bright‐field images further reveal the intimate interactions between cells and the hydrogel matrix walls (Figure 5c). Cells indicated by arrows in GelMA appear largely obstructed, exhibiting minimal intercellular connectivity, whereas in m‐GelMA, cells are observed traversing the matrix walls and establishing connections with cells residing in adjacent pores. This observation is corroborated by SEM analysis, which shows that cells cultured on the m‐GelMA hydrogels are more populated, and form better focal adhesions and denser intercellular connections compared to those on the GelMA counterparts (Figure 5d and Figure S23). The importance of optimal pore size for tissue regeneration has been well‐documented, and the hydrogel developed in this study exhibits structural characteristics conducive to fibroblast activity (5–15 µm), vascularized bone‐like tissue (15 µm), and skin tissue regeneration (20–125 µm) [54, 55, 56, 57]. Accordingly, the macroporous architecture significantly enhanced cell attachment and proliferation, demonstrating the potential of this platform for tissue engineering and disease modeling tailored to specific cell types. While these findings establish a clear phenotypic response to the engineered pore architecture, the underlying molecular mechanisms remain to be elucidated. Future transcriptomic and protein expression analysis could provide deeper insight into the signaling pathways governing cell‐material interactions and the observed cellular responses.

## 3 Conclusions

In summary, we have developed a GelMA‐based emulsion ink that enables the fabrication of highly interconnected porous hydrogel architectures through solvent‐phase removal, without compromising photopolymerization efficiency. Using DLP‐based vat polymerization 3D printing, the resulting hydrogels exhibit well‐connected pore networks and enhanced mechanical performance, with both pore architecture and mechanical properties readily tunable by adjusting the solvent content. This fabrication strategy is broadly applicable across a range of photocurable biopolymer‐based hydrogels, including ALMA, HAMA, and GelMA–alginate hybrid systems. Moreover, the macroporous structures promote improved cell attachment and proliferation, leading to denser and more integrated cellular networks. By enabling tunable microarchitecture and mechanical performance, this vat polymerization‐compatible strategy provides a versatile approach for fabricating functional hydrogel scaffolds for tissue engineering applications.

## 4 Experimental Section

### 4.1 Materials

Gelatin (from bovine skin, type B), alginic acid sodium salt (from brown algae, low viscosity), hyaluronic acid sodium salt (from *Streptococcus equi*), methacrylic anhydride (≥ 94%), 2‐aminoethyl methacrylate hydrochloride (90%), MES sodium salt (≥ 99%), Lithium phenyl‐2,4,6‐trimethylbenzoylphosphinate (≥ 95%), poly(ethylene glycol) (average Mw 1500), Rhodamine B (≥ 95%). To remove inhibitor, methacrylate anhydride was passed through a basic alumina column before use. All other reagents were used as received unless stated otherwise.

### 4.2 Instruments

The ^1^H NMR spectra were acquired on a Bruker Avance 400 MHz spectrometer at 24°C. The sonication was done through Vibra Cell Ultra Sonic Processor (Sonics VCX‐750) equipped with a 6 mm probe. The hydrodynamic sizes of solvent nanodroplets were measured in triplicate with Zetasizer Ultra (Malvern) that equipped with He–Ne laser (λ = 633 nm) at 24°C, and analyzed using ZS XPLOERE software (v2.0.1.1). The UV‐vis spectra were measured with a CARY 300 spectrophotometer (Agilent) and UVProbe software (v2.42). The geometries and. stl files were designed and generated using Autodesk Fusion, printing parameters like slicing thickness and cure time were set up using PhotonWorkshop (2.2.15, Anycubic). The 3D printing was performed with a DLP 3D printer (Photon Mono SE, Anycubic). The lyophilized samples were sputter with platinum with CCU‐010 compact coater (Safematic, GmbH). The morphology of the 3D printed hydrogels was observed with scanning electron microscope (SEM) on ΣIGMA‐HD (ZEISS) equipped with a hot field emission gun at an acceleration voltage of 1 kV. The fluorescent 3D images of hydrogels were acquired using a Nikon AX R NSPARC confocal with Plan Apo λS 25x Silicone NA 1.05 objective. The images were captured using 488 nm excitation and NSPARC SPPC super‐resolution detector. The swollen 3D scaffold was imaged with Xradia 515 Versa x‐ray Microscope (Zeiss). Compressive mechanical property was characterized using a MARK‐10 Universal test machine (IDM Instrument) with force gauge model M5‐20, the data was collected using MESUR Software (v2.0.6). The rheological and mechanical properties were characterized immediately after printing using an Anton Paar MCR502 Rheometer equipped with a Peltier system for temperature control, and the data was analyzed using RheoCompass (v1.32.258). The cell viability of the 3D‐printed was measured with a multimode plate reader (Tecan M200 Pro Infinite). The fluorescent images of cell cultured on the hydrogel surface were taken with Leica SP8 CLSM with PMT detector, and the tile scan was acquired with LAS X Navigator. The SEM samples with cells cultured on hydrogel were first dehydrated in Biowave Pro+ (Pelco), and lyophilized using Autosamdri‐815 A Critical Point Dryer (Tousimis).

### 4.3 Synthesis of GelMA, ALMA, and Hyaluronic Acid Methacrylate (HAMA)

Briefly, 10 g of gelatin (bovine, type B, Gelomics) was dissolved into 100 mL of Dulbecco's Phosphate‐Buffered Saline at 60°C until fully dissolved. Then, 0.6 mL of MAA (after inhibitor removal) was then added to the gelatin solution dropwise, and gently stirred at 60°C for 5 h. The solution was diluted in an equal volume of DPBS to quench the reaction, then centrifuged at 2000 rpm for 5 min to remove the precipitate. Afterwards, the diluted solution was transferred to a dialysis tube with 14 kDa cutoff to dialyze against deionized water (diH_2_O) for 7 days at 40°C, and the water was refreshed twice every day to remove the salt and unreacted MAA. Finally, the solution was freeze‐dried, and lyophilized GelMA was obtained and stored at room temperature.

Protocol for ALMA was adapted from Joen et al., [58].: 2 g of low‐molecular‐weight sodium alginate (Sigma Aldrich) was dissolved in a 50 mM 2‐morpholinoethanesulfonic acid (MES) buffer solution (1% w/v) containing 0.5 M NaCl (1% w/v), pH was adjusted to 6.5 using hydrochloric acid. N‐hydroxysuccinimide (NHS, 0.88 g; Sigma Aldrich) and 1‐ethyl‐3‐(3‐dimethylaminopropyl)‐carbodiimide hydrochloride (EDC, 2.905 g; Sigma Aldrich) were added to the mixture to activate the carboxylic acid groups of the alginate. After 10 min, 1.26 mL of 2‐aminoethyl methacrylate (AEMA, Sigma Aldrich) was added dropwise (molar ratio of NHS: EDC: AEMA = 1:2:1), and the reaction was maintained at room temperature for 48 h to allow maximum substitution. The mixture was precipitated with excess of acetone and then dried at a 40°C oven, and later rehydrated to a 1% w/v solution with deionized water. The solution was then dialyzed against deionized water for 10 days, with the water being refreshed twice a day, and subsequently lyophilized to obtain ALMA.

Synthesis of HAMA is adapted from Zhang et al., and Hachet et al., [59, 60]. Briefly, 4 g of hyaluronic acid sodium salt (M_w_: 1.3–1.8 MDa, cat. 53747, Sigma Aldrich) was dissolved in 400 mL of de‐ionized water at 4°C for continuous stirring overnight. Then, 400 mL of DMF was added dropwise to the solution, and MAA (4.64 g) was added to the solution mixture dropwise, with vigorous stirring. The pH was monitored and adjusted to 8–9 using 1 M NaOH for an hour. The reaction was leave on at 4°C overnight. NaCl was dissolved to reach a concentration of 0.5 M, and the product was precipitated by adding an excess amount of ethanol and leave in ‐80°C to decrease the solubility of HAMA in water. The supernatant was then removed. The white precipitate was dissolved in de‐ionized water and purified via dialysis against water for 7 days, with water being refreshed twice a day. The HAMA was recovered by freeze‐drying and later stored at room temperature before use.

### 4.4 Preparation of Nanodroplet Containing Resin

The resin was prepared as described in Table S2 in advance of the experiments and stored at 4°C. Specifically, three precursor solutions were prepared by separately dissolving lyophilized GelMA (7.5%, w/v), ALMA (2%, w/v), and HAMA (1%, w/v) in deionized water containing 2% (v/v) Tween‐20. A fixed concentration of 5% of photoinitiator lithium pheyl‐2,4,6‐trimethylbenzoylphosphonate (LAP, Sigma Aldrich) was then added to the resin. Before use, the GelMA resin was warmed in a 50°C oven. The organic solvents (chloroform or dichloromethane) at different volume percentages were then added to the resin, and the resin solution was sonicated with probe in a water bath at room temperature for 10 min (20% power amplitude). The in situ photopolymerization kinetics study on emulsion resin was performed with an Anton Paar MCR‐502 modular compact rheometer equipped with a 15 mm steel upper plate and an acrylic lower base in a parallel plate configuration at 37°C. The initial gap was 0.1 mm with the instrument set to maintain 0 ± 0.1N of axial force. A strain of 0.05% and an angular frequency of 10 rad/s was constantly applied for all measurements. The UV irradiation (intensity: 1.68 mW cm^−^^2^) was switched on 30 s after the test began. Shear viscosity measurements of resins were carried out in a steady‐state mode at 37°C, and the shear rate range was set from 1 to 1000 s^−1^. The diameter of the geometry and the gap between the two geometries were 25 and 0.1 mm, respectively.

### 4.5 Preparation of 3D‐Printed Porous Hydrogel

The prepared emulsion resin was transferred to the 3D printer vat. During printing process, the resin was subsequently irradiated with controlled ultraviolet light (λ_max_  =  405 nm) with a light intensity of approximately 1.68 mW cm^−^^2^. The targeted object dimension was designed specifically for compressive (disk: diameter × thickness  =  10 × 3 mm) and rheological testing (disk: diameter × thickness = 35 × 3 mm) and the. stl files were generated with Autodesk Fusion, and the printing parameters such as exposure time per layer, slicing thickness and speed were determined on the Photon workshop. The first 4 layers were exposed to irradiation for 30s. The cure time for the rest of the layers were stated in the table supplied. The Z lift distance was 6 mm, the Z lift speed was 4 mm/s, and the Z retract speed was 6 mm/s. After printing, the hydrogel was carefully washed with diH_2_O, removed with scrapper, and allowed for 10 min of post‐curing for maximum and uniformed polymerization across samples.

### 4.6 Morphological Characterization of Macroporous Hydrogel

SEM was conducted to examine the structural morphology of the macroporous hydrogels, samples were dialyzed in diH_2_O after printed. Then, they were snap frozen with liquid nitrogen, fractured to reveal the inner microarchitecture and freeze‐dried. The lyophilized samples were sputter with platinum and observed with SEM (ΣIGMA‐HD, ZEISS) at an acceleration voltage of 1 kV.

For confocal fluorescence imaging, the printed hydrogel was immersed in a 0.001 g/mL rhodamine B solution for 24 h, followed by lyophilization. The processed hydrogel was then placed in a glass bottom well plate for imaging.

The swollen scaffold morphology in 3D was investigated by micro‐CT analysis. Micro‐CT scans were acquired using Zeiss Xradia 515 Versa x‐ray Microscope at a voxel resolution of 1 µm. Prior to imaging, swollen samples were contrast‐enhanced using an aqueous copper(II) sulfate solution and scanned within 24 h of staining. Cross‐sectional images were reconstructed from the acquired projections, and 3D visualization was performed using Avizo (Thermo Fisher).

### 4.7 Mechanical Properties Studies

The uniaxial compression tests were performed to hydrogel samples at room temperature. The hydrogel samples were printed into a disk (diameter × thickness  =  10 × 3 mm). The rate of displacement was set constant as 1.1 mm/min, and the samples were compressed until fracture. The compressive modulus was calculated from the slope value at 1%–4% strain of the stress‐strain curve, the toughness was obtained from the area under the stress–strain curve.

Viscoelastic behavior of the hydrogels was done using an Anton Paar MCR‐502 modular compact rheometer with a 50 mm steel parallel plate geometry at room temperature. The hydrogel samples were printed into disk (diameter × thickness  =  35 × 3 mm) for rheological performance tests. Sandpaper (240 grit) was glued to the surface of both top and bottom plates to prevent the slip on the wall, and a normal force of 1N was applied initially in all tests to hold the sample in place. The strain sweep tests were performed in oscillation mode for characterization of linear viscoelastic region and the dynamic yielding behavior of the material, in which the shear amplitude varies from 0.1% to 100% at a constant frequency of 10 rad/s. Frequency sweep tests were performed between 100 to 1 rad/s at 5% shear strain within the linear viscoelastic region.

### 4.8 Swelling

10‐fold excess of amount of diH_2_O or DPBS solutions were added to the lyophilized hydrogels, and the samples were allowed to swell in the solutions at 25°C (diH_2_O) and 37°C (DPBS). The surface of the hydrogel was gently blotted with Kimwipe for the removal of excess solution before weighing, the weights of hydrogel samples were measured at corresponding time points over 72 h, and the measurements were done in triplicate. The swelling ratio was calculated using the Equation (1):

(1) $$S_{W}\left(\%\right)=\frac{W_{t}-W_{i}}{W_{i}}\;\times 100\%$$

where *W_t_* denotes the weight of swollen hydrogel at time t and W_i_ represents the initial weight of lyophilized hydrogel.

### 4.9 In Vitro Cytotoxicity

The cytotoxicity of macroporous hydrogel was examined via CCK8 assay. In brief, HEK‐293 was seeded in a 24‐well plate at a density of 5000 cells/well 24 h prior to the experiment. Hydrogel samples printed with Group 3 resin were lyophilized. Before the experiments, samples were immersed in ethanol for 10 min for sterilization, followed by culture medium immersion for 30 min. The samples were then cocultured with cells for 1/3/5 day(s), and the control group contains no hydrogel but the cells alone. Afterward, 100 µL CCK8 reagent (Biorad) was added to each well, and the absorbance was measured at 460 nm after 3 h incubation by Tecan M200 Pro Infinite plate reader. All the experiments were carried out in triplicate to ensure the validity of the result.

### 4.10 Cell Attachment Analysis

Following printing, the constructs were immersed in water for 2 h to facilitate partial extraction of chloroform from the hydrogel matrix, followed by freeze‐drying to remove residual solvent and preserve the porous architecture prior to cell culture. Freeze‐dried hydrogel scaffold samples were sterilized in 70% ethanol. After 30 min, samples were transferred to full medium and leave overnight to fully displace the ethanol and reach swelling equilibrium. Suspended hCMEC/D3 cells (GFP‐positive) or MDA‐MB‐231 cells (GFP‐positive) were then seeded on the samples at a density of 20 000 cells/well in a 24‐well plate. The hCMEC/D3 cells were cultured in basal medium supplemented with recombinant human EGF (rhEGF), L‐Glutamine, hydrocortisone, heparin sulfate, ascorbic acid and 2% fetal bovine serum (FBS), as recommended by the manufacturer. The MDA‐MB‐231 cells were grown in DMEM/F12 containing 10% FBS. The medium was replaced daily to avoid additional variability, and the cells were imaged at day 1, 3 and 5 using Leica SP8 CLSM, images were taken with tile scanning to obtain larger field of view. Fluorescent intensity was measured from the images. The cells cultured on day 3 and 5 were fixed with 4% paraformaldehyde and stained with Hoechst 33342 and ActinRed 555 ReadyProbes (Thermo Fisher) for close‐up morphology analysis. Cell viability was assessed using a live/dead viability assay. Briefly, on day 5, samples were rinsed gently with PBS and incubated in a staining solution containing calcein AM and ethidium homodimer‐1 in PBS for 30 min at room temperature in the dark. Following incubation, samples were imaged immediately using CLSM. The SEM samples were fixed with 2.5% glutaraldehyde, and dehydrated with graded ethanol series (50%/ 70%/ 90% /100%/ 100%) in Biowave, lyophilized using ScCO_2_ critical point dryer and sputtered with a 10 nm platinum layer for SEM observations.

### 4.11 Statistical Analysis

All the results were repeated in triplicate. The results are displayed as means ± standard deviation (SD), and *p* < 0.05 was considered to be statistically significant. Statistical analyses were performed using GraphPad Prism (version 10.1.2). Significance was denoted as follows: *p* > 0.05, not significant (ns); *p* ≤ 0.05 (*); *p* ≤ 0.01 (**); *p* ≤ 0.001 (***); and *p* ≤ 0.0001 (****).

## Author Contributions

Ruirui Qiao and Liwen Zhang conceived the idea and supervised this project. Liwei Liu designed and performed the experiments, analyzed the data and drafted the manuscript. Xumin Huang helped with electron microscopy imaging. Yuan Xu assisted with rheology, Jason R. Stokes provided the rheometer. Jinghan Qin, Yi Du, and Dandan Cui performed the micro‐CT. Xiaoming Sun provided support in fluorescence microscopy. Ruirui Qiao, Liwen Zhang, Yuan Xu, Xumin Huang, Xinyan Gu, Naufal Kabir Ahamed Nasar, Jiali Zhai, and Thomas P. Davis provided critical feedback and revised the manuscript.

## Conflicts of Interest

The authors declare no conflicts of interest.

## Supporting information




**Supporting File**: smtd70988‐sup‐0001‐SuppMat.docx.

## Data Availability

The data that support the findings of this study are available from the corresponding author upon reasonable request.
