# Supplementary material for: Vat Polymerization 3D Printing of Hydrogels With Tunable Porosity
Source: Small Methods. 2026 Aug 28;10(19):e70988. doi: 10.1002/smtd.70988 (PMC13646038; doi:10.1002/smtd.70988)
Supplement: Supplementary file 1 [file SMTD-10-e70988-s001.docx]

Supporting Information

**Vat Polymerization 3D Printing of Hydrogels with Tunable Porosity**

Liwei Liu, Liwen Zhang*, Xumin Huang, Yuan Xu, Jason R. Stokes, Jinghan Qin, Yi Du, Dandan Cui, Xinyan Gu, Naufal Kabir Ahamed Nasar, Xiaoming Sun, Jiali Zhai, Thomas P. Davis and Ruirui Qiao*

L. Liu, Dr. L. Zhang, Dr. X. Huang, X. Gu, N. K. A. Nasar, Prof. T. P. Davis, A/Prof. R. Qiao

Australian Institute for Bioengineering and Nanotechnology, The University of Queensland, Brisbane, QLD 4072, Australia.

Email: [liwen.zhang@uq.edu.au](mailto:liwen.zhang@uq.edu.au); [r.qiao@uq.edu.au](mailto:r.qiao@uq.edu.au)

Dr. Y. Xu, Prof. J. R. Stokes

School of Chemical Engineering, The University of Queensland, Brisbane, QLD 4072, Australia

J. Qin, Prof. Y. Du, A/Prof. D. Cui

School of Physics, Beihang University, Beijing 100191, P. R. China

Centre of Quantum and Matter Sciences, International Research Institute for Multidisciplinary Science, Beihang University, Beijing 100191, P. R. China

Dr. X. Sun

Australian National Fabrication Facility Queensland Node (ANFF-Q), The University of Queensland, Brisbane, QLD 4072, Australia

A/Prof. J. Zhai

School of Science, STEM College, RMIT University, Melbourne, VIC 3000, Australia

Keywords: Macroporous hydrogel, Soft Materials, 3D printing, Cell proliferation, Tissue engineering

Figure S1. ^1^H NMR spectra of gelatin and gelatin methacrylate in D_2_O. Characteristic peaks (vinyl groups at 5.20-5.70 ppm and lysine group at 1.75 ppm) were marked with dash lined boxes, aromatic peaks were used as reference. The degree of methacrylation was quantified as 70%.

Figure S2. Droplet size distribution of a mixture of chloroform and a 7.5% (w/v) GelMA and 2% (w/v) PEG solution (at a volume ratio of 2:10), without surfactant and with two different surfactants (CTAB and Tween 20), after sonication for 5 and 10 minutes. Data represents the mean ± SD (n = 3 independent samples), error bars indicate SD.

Figure S3. Photographs of emulsion resins prepared with different solvent-to-aqueous phase volume ratios (aqueous phase: 7.5% GelMA (w/v) and 2% PEG (w/v)) 20 min after probe sonication. The leftmost image shows the solution before sonication. Black arrows indicate phase separation of the organic solvent, demonstrating emulsion instability at higher solvent contents.

**0 hr**

**24 hr**

Figure S4. Droplet size distribution of a mixture of chloroform and a 7.5% (w/v) GelMA and 2% (w/v) PEG solution (at a volume ratio of 2:10) at 0 and 24 hr after sonication.

Figure S5. Three CAD models designed to demonstrate printability of GelMA emulsion resin in fabricating complex structure, with geometries indicated on the graph.

Figure S6. A comparison between this work and the reported porous hydrogel-based materials in terms of the pore size. References are listed in Table S3.

Figure S7. Swelling profile of macroporous hydrogel immersed in water at 37°C. Data represents the mean ± SD (*n* = 3 independent samples), error bars indicate SD.

Figure S8. Swelling profile of macroporous hydrogel immersed in PBS (pH 7.4) at 37°C. Data represents the mean ± SD (*n* = 3 independent samples), error bars indicate SD.

Figure S9. 3D micro-CT reconstruction of Group 3 porous hydrogel. (Left) Segmented pore network (blue) within the reconstructed volume. (Right) Corresponding volume rendering showing pore morphology and spatial distribution. Scale bar: 20 μm.

Figure S10. Amplitude independence of the storage modulus (G’) and loss modulus (G’’) for different hydrogel samples.

Figure S11. The shear stress-strain curves of macroporous GelMA hydrogel different nanodroplet (solvent) volume fractions.

Figure S12. The compressive stress-strain curves of macroporous GelMA hydrogel with different nanodroplet (solvent) volume fractions, three individual tests were done for result validation.

Figure S13. ^1^H NMR spectra of sodium alginate and alginate methacrylate in D_2_O. Characteristic peaks (vinyl groups at 5.20-6.30 ppm and methyl group at 1.70-2.10 ppm) were labelled with dash lined boxes. The degree of methacrylation was quantified as 8%.

Figure S14. ^1^H NMR spectra of hyaluronic acid and hyaluronic acid methacrylate in D_2_O. Characteristic peaks (vinyl groups at 5.50-6.30 ppm and methyl groups at 1.70-2.10 ppm) were labelled with dash lined boxes. The degree of methacrylation was quantified as 18.01%.

Figure S15. The viscosity versus shear rate behavior for different resin formulations at 37°C.

Figure S16. Droplet size distribution of a 2% ALMA, 1% HAMA and 7.5% GelMA – 2% Alg emulsion resins (w/v) at 0 and 24 hr after sonication, the solvent fraction is 3:10 (v/v) solvent: aqueous solution in all samples. Data represents the mean ± SD (*n* = 3 independent samples), error bars indicate SD.

Figure S17. Photographs of 3D-printed ALMA, HAMA and GelMA-Alg hydrogels and their counterpart macroporous objects with different formulations. The CAD design is shown on the left.

Figure S18. The compressive stress-strain curves of conventional and macroporous hydrogels made of different hydrogel formulation (ALMA, HAMA and GelMA-alginate hybrid), three individual tests were done for result validation.

Figure S19. ^1^H NMR spectrum of 3D-printed m-GelMA in D₂O. No detectable resonance corresponding to chloroform (~7.26 ppm) was observed, indicating the effective removal of residual chloroform after processing.

Figure S20. Cytotoxicity of m-GelMA hydrogels towards HEK-293 cells with incubation periods of 1/3/5 day using CCK8 assay, in which the control group is treated without any hydrogel. Data represents the mean ± SD (*n* = 3 biological replicates), error bars indicate SD. The statistical significance was examined using two-way ANOVA with Šídák’s multiple-comparisons test.

Figure S21. Microscopic images of HEK-293 cells incubated with m-GelMA hydrogels treated in the incubation periods of 1/3/5 day, in which the control group is treated without any hydrogel. Scale bar: 10 µm.

Figure S22. Representative confocal laser scanning microscopic images of MDA-MB-231 cell morphology cultured on the GelMA and m-GelMA after 3 (left 2 columns) and 5 (right two columns) days of culture. The nucleus of the cell is depicted in blue, and the F-actin of the cell is depicted in red. Scale bar: 20 µm.

Figure S23. Representative scanning electron microscopic images of MDA-MB-231 cell morphology cultured on the GelMA and m-GelMA hydrogel scaffolds after 3 and 5 days of culture. Scale bar: 100 µm. The insets demonstrated the interconnection of the cells and how their shape adapted to the hydrogel matrix. Scale bar: 20 µm.

**Supplementary Table:**

| Resin | Surfactant | | Sonication | Solvent | | Droplet | |
| --- | --- | --- | --- | --- | --- | --- | --- |
|  |  | |  |  | |  | |
| GelMA: PEG  (w/v, %) | Surfactant | Surfactant:aqueous solution (v/v, %) | Time (min) | Solvent | Solvent: aqueous solution (v/v) | Droplet size (d.nm) | PDI^1^ |
| 7.5 : 2 | - | - | 5 | CHCl_3_ | 2:10 | 3115.1 ± 525.2 | 0.75 |
| 7.5 : 2 | - | - | 10 | CHCl_3_ | 2:10 | 3299.8 ± 603.3 | 0.88 |
| 7.5 : 2 | CTAB | 2 | 5 | CHCl_3_ | 2:10 | 459.0 ± 74.7 | 0.48 |
| 7.5 : 2 | CTAB | 2 | 10 | CHCl_3_ | 2:10 | 1157.6 ± 364.0 | 0.48 |
| 7.5 : 2 | Tween 20 | 2 | 5 | CHCl_3_ | 2:10 | 245.0 ± 11.7 | 0.22 |
| 7.5 : 2 | Tween 20 | 2 | 10 | CHCl_3_ | 2:10 | 208.7 ± 23.3 | 0.23 |

Table S1. Surfactant optimization for stable resin

^1^PDI: polydispersity

Table S2. 3D printing emulsion resin formulation

| Group | Resin | Solvent | | Droplet | |
| --- | --- | --- | --- | --- | --- |
|  | GelMA : PEG : LAP : Tween-20 | Solvent | Solvent:aqueous solution (v/v) | Droplet size (d.nm) | PDI |
| Control | 7.5 : 2 : 0.5 : 2 | - | - | - | - |
| 1 | 7.5 : 2 : 0.5 : 2 | CHCl_3_ | 1:10 | 272.9 ± 4.62 | 0.21 |
| 2 | 7.5 : 2 : 0.5 : 2 | CHCl_3_ | 2:10 | 342.81 ± 3.32 | 0.18 |
| 3 | 7.5 : 2 : 0.5 : 2 | CHCl_3_ | 3:10 | 308.06 ± 4.84 | 0.15 |
| ALMA : LAP : Tween-20 | | |  |  |  |
| 4 | 2 : 0.5 : 2 | - | - | - | - |
| 5 | 2 : 0.5 : 2 | CHCl_3_ | 3:10 | 789.6 ± 107.1 | 0.20 |
| HAMA : LAP : Tween-20 | | |  |  |  |
| 6 | 1 : 0.5 : 2 | - | - | - | - |
| 7 | 1 : 0.5 : 2 | CHCl_3_ | 3:10 | 2471 ± 91.4 | 0.12 |
| GelMA : Alginate : LAP : Tween-20 | | |  |  |  |
| 8 | 7.5 : 1 : 0.5 : 2 | - | - | - | - |
| 9 | 7.5 : 1 : 0.5 : 2 | CHCl_3_ | 3:10 | 637.6 ± 48.16 | 0.21 |

Table S3. Pore size of polymer materials

| Polymer materials | Form | Dispersity | Pore size range (µm) | Ref |  |
| --- | --- | --- | --- | --- | --- |
| PETMP/DiPETMP^1^ | Foam | Monodisperse | 107 ± 8 — 173 ± 15 | [1] |  |
| 2-Hydroxyethl Methacrylate | Monolith | Polydisperse | 0.6 - 18 | [2] |  |
| PEGDA^2^ | Hydrogel | Polydisperse | 122.0 ± 16.8 — 233.1 ± 51.5 | [3] |  |
| PVA/BaG^3^/Chitosan | Scaffold | Polydisperse | 10 — 600 | [4] |  |
| GelMA^4^ | Hydrogel | Polydisperse | 24.8 ± 10.3 — 58.7 ± 24.9 | [5] |  |
| Gelatin | Foam | Monodisperse | 80 — 800 | [6] |  |
| PEGDA | Foam | Mono-, bi- and tridisperse | 400 — 700 | [7] |  |
| GelMA | Hydrogel | Polydisperse | 70.68 ± 15.6 — 513.63 ± 39.88 | [8] |  |
| GelMA | Liquid foam | Monodisperse | 661 ± 41 — 1797 ± 41 | [9] |  |
| Gelatin | Scaffold | Polydisperse | 280 — 550 | [10] |  |
| Alginate | Hydrogel | Polydisperse | 0.76 ± 0.06 — 6.4 ± 0.6 | [11] |  |
| Chitosan | Foam | Polydisperse | 100 — 500 | [12] |  |

^1^Pentaerythritol tetramercaptopropionate and dipentaerythritol hexakis(3-mercaptopropionate)

^2^Poly(ethylene glycol) Diacrylate

^3^Bioactive glass

^4^Gelatin methacrylate

Table S4. 3D printed object parameter - ALMA/HAMA/GelMA-alginate hydrogel

| # | Polymer | Solvent: aqueous solution (v/v) | 3D-printing parameter | | 3D-printed object | |
| --- | --- | --- | --- | --- | --- | --- |
|  |  |  | Curing time/layer (s) | Thickness/  layer (mm) | Diameter (mm) | Thickness (mm) |
| 1 | ALMA | - | 10 | 0.05 | 11.26 ± 0.04 | 3.05 ± 0.01 |
| 2 | m-ALMA | 3:10 | 15 | 0.05 | 11.24 ± 0.01 | 3.02 ± 0.02 |
| 3 | HAMA | - | 20 | 0.05 | 11.13 ± 0.02 | 3.04 ± 0.02 |
| 4 | m-HAMA | 3:10 | 30 | 0.05 | 10.96 ± 0.01 | 2.93 ± 0.12 |
| 5 | GelMA-Alg | - | 10 | 0.05 | 11.38 ± 0.11 | 3.07 ± 0.03 |
| 6 | m-GelMA-Alg | 3:10 | 30 | 0.05 | 11.44 ± 0.24 | 3.09 ± 0.03 |

**Reference:**

[1]	D. N. Amato, D. V. Amato, M. Sandoz, J. Weigand, D. L. Patton, C. W. Visser, *ACS Applied Materials & Interfaces* **2020**, *12*, 42048-42055.

[2]	S. Kovačič, D. Štefanec, P. Krajnc, *Macromolecules* **2007**, *40*, 8056-8060.

[3]	S. Sokic, M. Christenson, J. Larson, G. Papavasiliou, *Macromol. Biosci.* **2014**, *14*, 731-739.

[4]	H. S. Mansur, H. S. Costa, *Chem. Eng. J.* **2008**, *137*, 72-83.

[5]	G.-L. Ying, N. Jiang, S. Maharjan, Y.-X. Yin, R.-R. Chai, X. Cao, J.-Z. Yang, A. K. Miri, S. Hassan, Y. S. Zhang, *Adv. Mater.* **2018**, *30*, 1805460.

[6]	M. Costantini, J. Jaroszewicz, Ł. Kozoń, K. Szlązak, W. Święszkowski, P. Garstecki, C. Stubenrauch, A. Barbetta, J. Guzowski, *Angew. Chem. Int. Ed.* **2019**, *58*, 7620-7625.

[7]	C. W. Visser, D. N. Amato, J. Mueller, J. A. Lewis, *Adv. Mater.* **2019**, *31*, 1904668.

[8]	Z. Luo, G. Tang, H. Ravanbakhsh, W. Li, M. Wang, X. Kuang, C. E. Garciamendez-Mijares, L. Lian, S. Yi, J. Liao, M. Xie, J. Guo, Z. Zhou, Y. S. Zhang, *Adv. Mater.* **2022**, *34*, 2108931.

[9]	L. Carballido, M. L. Dabrowski, F. Dehli, L. Koch, C. Stubenrauch, *Journal of Colloid and Interface Science* **2020**, *568*, 46-53.

[10]	S. A. Poursamar, J. Hatami, A. N. Lehner, C. L. da Silva, F. C. Ferreira, A. P. M. Antunes, *Materials Science and Engineering: C* **2015**, *48*, 63-70.

[11]	J. P. K. Armstrong, M. Burke, B. M. Carter, S. A. Davis, A. W. Perriman, *Advanced Healthcare Materials* **2016**, *5*, 1724-1730.

[12]	A. Barbetta, A. Carrino, M. Costantini, M. Dentini, *Soft Matter* **2010**, *6*, 5213-5224.
